# The application of gradient dose segmented analysis of in‐vivo EPID images for patients undergoing VMAT in a resource‐constrained environment

**DOI:** 10.1002/acm2.13985

**Published:** 2023-04-13

**Authors:** Christoffel Jacobus van Reenen, Christoph Jan Trauernicht, Casey Bojechko

**Affiliations:** ^1^ Department of Medical Imaging and Radiation Oncology Medical Physics Division Stellenbosch University Cape Town Western Cape South Africa; ^2^ Department of Radiation Medicine and Applied Sciences University of California San Diego, La Jolla San Diego California USA

**Keywords:** Halcyon, in‐vivo EPID images, quality assurance

## Abstract

The gamma analysis metric is a commonly used metric for VMAT plan evaluation. The major drawback of this is the lack of correlation between gamma passing rates and DVH values. The novel GDSA_mean_ metric was developed by Steers et al. to quantify changes in the PTV mean dose (D_mean_) for VMAT patients. The aim of this work is to apply the GDSA retrospectively on head‐and‐neck cancer patients treated on the newly acquired Varian Halcyon, to assess changes in GDSA_mean_, and to evaluate the cause of day‐to‐day changes in the time‐plot series. In‐vivo EPID transmission images of head‐and‐neck cancer patients treated between August 2019 and July 2020 were analyzed retrospectively. The GDSA_mean_ was determined for all patients treated. The changes in patient anatomy and rotational errors were quantified using the daily CBCT images and added to a time‐plot with the daily change in GDSA_mean_. Over 97% of the delivered treatment fractions had a GDSA_mean_ < 3%. Thirteen of the patients received at least one treatment fraction where the GDSA_mean_ > 3%. Most of these deviations occurred for the later fractions of radiotherapy treatment. Additionally, 92% of these patients were treated for malignancies involving the larynx and oropharynx. Notable deviations in the effective separation diameters were observed for 62% of the patients where the change in GDSA_mean_ > 3%. For the other five cases with GDSA_mean_ < 3%, the mean pitch, roll, and yaw rotational errors were 0.90°, 0.45°, and 0.43°, respectively. A GDSA_mean_ > 3% was more likely due to a change in separation, whereas a GDSA_mean_ < 3% was likely caused by rotational errors. Pitch errors were shown to be the most dominant. The GDSA_mean_ is easily implementable and can aid in scheduling new CT scans for patients before significant deviations in dose delivery occur.

## 1 INTRODUCTION

The World Health Organization estimates that approximately 70% of deaths from cancer occur in low‐and middle‐income countries.^1^ In South Africa (an upper middle‐income country^2^), approximately 107 467 new cancer cases were reported in 2018 alone, along with 57 373 cancer deaths.^3^ Roughly 61% of these patients undergo external beam radiation therapy as part of their treatment regime.^4^ Radiotherapy departments should have quality assurance (QA) programs in place, to ultimately ensure a high accuracy of treatment dose delivery for all patients.

In addition to patient setup verification, electronic portal imaging devices (EPIDs) have been in routine use to perform various quality control (QC) procedures in radiotherapy. These include multi‐leaf collimator (MLC) tests and offline, pre‐treatment patient‐specific QC procedures for volumetric modulated arc radiotherapy (VMAT) and intensity modulated radiotherapy (IMRT) treatments.^5^, ^6^, ^7^ A variety of EPID‐based pre‐treatment verification methods have been described in literature; the acquisition can be classified as either non‐transmission pre‐treatment dosimetry, non‐transmission treatment dosimetry, or transmission treatment dosimetry.^6^ In addition to the various modes of acquisition, the delivered dose can be estimated using several different approaches, including predicted forward‐projected EPID comparisons and simple back‐projection of measured data.^6^, ^8^, ^9^, ^10^

It is becoming increasingly popular to use transmission EPID‐based dosimetry to verify that the patient's received dose is correct and multiple commercial systems are now available for use.^6^, ^8^, ^11^, ^12^ One such approach is to use the 3D reconstructed EPID dose to calculate dose‐volume histogram (DVH) statistics in the planning computed tomography (pCT) dataset. The calculation also approximates all tissues to water, which in itself does not represent the true dose delivery to the patient.^8^ Other commercial approaches allow users to compare first‐fraction EPID transmission images to those of all subsequent fractions by means of applying the usual gamma‐analysis metric. Although these are useful metrics to quantify the repeatability of treatment fractions, these methods do not provide DVH‐specific statistics that relate the delivered dose to the planning target volume (PTV).^6^, ^8^, ^9^, ^12^, ^13^, ^14^

To address this issue, a new analysis technique was introduced as a pre‐treatment verification method in a doctoral dissertation that did not require recalculation of EPID‐based data to patient dose, or analysis by means of the pass/fail gamma criteria.^15^ Recently, Steers et al.^16^ published their work on applying the gradient dose segmented analysis (GDSA) technique to in‐vivo EPID images for dose verification. Their results showed that the GDSA could successfully predict changes in the PTV mean dose (D_mean_), a clinically relevant dosimetric endpoint.^16^

The aim of this work is to apply the GDSA analysis method described by Steers et al. to determine the change in GDSA_mean_ for head‐and‐neck cancer patients treated with VMAT, and to identify changes in treatment where the GDSA exceeds a 3% threshold. The GDSA_mean_ threshold is based on the recommended dose delivery accuracy tolerance level of 3%.^17^ This data was used to determine the treatment quality of following the implementation of VMAT for one year, and will serve as a baseline for subsequent review.

## 2 MATERIALS AND METHODS

### 2.1 Retrospective EPID image data collection

All the EPID images used in this study were acquired on a Varian Halcyon v2.0 linear accelerator, which comes equipped with a 43 cm x 43 cm aS1200 megavoltage imaging panel (Varian Medical Systems, Palo Alto, CA). The panel is mounted directly opposite the single 6xFFF MV beam at a source‐to‐imager distance (SID) of 154 cm, which corresponds to a 28 cm x 28 cm projection at 100 cm source‐axis distance (SAD). The EPID continuously integrates the obtained signal from the entire treatment field during arc treatments. The individually acquired transit images are then automatically exported on an arc‐by‐arc basis to the ARIA record‐and‐verify system (Varian Medical Systems, Palo Alto, CA). The EPID calibration workflow follows a semi‐automated step‐by‐step approach, where dark field and flood field corrections are applied. Thereafter, the EPID is calibrated in terms of Calibrated Units (CU), where 1 CU is equivalent to 1 MU for a standard 10 cm x 10 cm field size. The linear accelerator's output is verified prior to starting the EPID calibration workflow by following the IAEA TRS‐398 code of practice. In addition to monthly output checks, the QA includes the weekly delivery of standard field sequences in QA mode to check the constancy of the EPID response. The Halcyon is also equipped with an automated machine performance check (MPC) which verifies the daily machine output and its drift, along with other parameters.

Approval was granted by the institutional Health Research Ethics Committee (HREC) for the study to proceed. The EPID images were obtained for all head‐and‐neck cancer radiotherapy patients treated between August 2019 and July 2020. This marked the first year of the simultaneous introduction of the Varian Halcyon, as well as VMAT treatments in our Radiation Oncology Division.

### 2.2 Retrospective kV CBCT image data collection

In addition to the MV imaging capabilities, the Halcyon is also equipped with a kilovoltage cone‐beam computed tomography (kV CBCT) imaging system. All patients treated with VMAT were set up by matching the daily kV CBCT images to pCT images for all treatment fractions. After the acquisition of the daily setup image, the system performs an automated online matching between the kV CBCT setup image and the pCT. A team of qualified and trained radiotherapists then verify the image matching and the appropriate couch corrections are applied before treatment. The kV CBCT image and matching with the pCT is automatically exported to the record‐and‐verify system after treatment.

The kV CBCT images and registration matrices were obtained for all the head‐and‐neck cancer radiotherapy patients treated within the study period.

### 2.3 EPID image analysis using the GDSA algorithm

The collected EPID images were analyzed by running an in‐house script in MATLAB R2019a (The MathWorks, Inc., Natick, MA) using the GDSA method formulated by Steers et al.^15^, ^16^ In summary, the GDSA method takes the acquired EPID reference composite image set for the first treatment fraction as an input, and uses the subsequent treatment fraction composite images as the comparison datasets. The dose gradient map is computed using the normalized composite of the reference EPID images. The dose differences between the reference and comparison composite datasets are then calculated and normalized to the dose maximum in the reference dataset. The dose distributions are then segmented into different regions of interest, based on a set dose threshold of 5% and dose‐gradient threshold of 3%/mm relative to the reference dataset. This relationship is described by Moran et al.^18^ in Equation (1):

(1) $$G_{i}=\sqrt{\sum {\left(\frac{\Delta d_{ij}}{\Delta x_{ij}}\right)}^{2}}\;$$

where $G_{i}$ is the generalized gradient at a given pixel, *i*, $\Delta d_{ij}$ is the dose difference between the pixel *i* and its four nearest neighbors, *j*, and $\Delta x_{ij}$ is the distance between *i* and *j*.

For the Varian Halcyon, $\Delta x_{ij}\;\approx$ 0.336 mm, which corresponds to the EPID pixel spacing based on the physical dimensions of the imager panel (43 cm x 43 cm) and image matrix size (1280 × 1280 pixels). The mean percent dose difference in the high‐dose, low‐gradient regions of the composite distributions has been shown to be a predictor for changes in the PTV D_mean_.^15^, ^16^ This normalized mean dose difference in the high‐dose region is referred to as the GDSA_mean_, abbreviated as GDSA_μ_ (%).^15^, ^16^

The standard deviation of the GDSA_μ_ was calculated for each treatment fraction as the standard deviation of the distribution of pixels in the high‐dose low‐gradient region of interest.

### 2.4 kV CBCT image analysis: Patient separation

After the EPID images were analyzed using the GDSA, the kV CBCT images of patients where the |GDSAμ|≥3% were inspected. The anterior‐posterior (A‐P) and lateral separations were measured as the absolute change in the outline of the body contour across the treatment isocenter slice of the kV CBCT for all treatment fractions. The separations were reported as the absolute difference between the reference separation and the separation from subsequent fractions for the A‐P direction according to Equation (2):

(2) $$\Delta d_{A-P,\;n}\;\left(cm\right)=\;\left|{\left(d_{A-P}\right)}_{ref}-\;{\left(d_{A-P}\right)}_{n}\right|$$

where $\Delta d_{A-P,n}$ is the calculated change in A‐P separation (in cm) for the *n* ^th^ fraction, ${\left(d_{A-P}\right)}_{ref}$ is the A‐P separation on the treatment isocenter slice of the reference kV CBCT, and ${\left(d_{A-P}\right)}_{n}$ is the A‐P separation on the treatment isocenter slice of the *n* ^th^ subsequent kV CBCTs.

Similarly, the absolute difference for the lateral dimensions were calculated according to Equation (3) as:

(3) $$\Delta d_{lat,\;n}\;\left(cm\right)=\;\left|{\left(d_{lat}\right)}_{ref}-\;{\left(d_{lat}\right)}_{n}\right|$$

where $\Delta d_{lat,n}$ is the calculated change in lateral separation (in cm) for the *n* ^th^ fraction, ${\left(d_{lat}\right)}_{ref}$ is the lateral separation on the treatment isocenter slice of the reference kV CBCT, and ${\left(d_{lat}\right)}_{n}$ is the lateral separation on the treatment isocenter slice of the *n* ^th^ subsequent kV CBCTs.

The effective separation change diameter, $\Delta d_{eff,\;n}\left(cm\right)$, was then computed using Equation (4):

(4) $$\Delta d_{eff,n}\;\left(cm\right)=\sqrt{\Delta d_{lat,n}\cdot \Delta d_{A-P,n}}\;$$

where $\Delta d_{lat,n}$ is the lateral separation change (cm) and $\Delta d_{A-P,n}$ is the A‐P separation change (cm) for the *n* ^th^ fraction.^19^

### 2.5 kV CBCT image analysis: Rotational set‐up corrections

The kV CBCT images were then analyzed in the image registration workspace of the record‐and‐verify system and the rotational corrections were computed for the pitch (θ), roll (ζ), and yaw (φ). This was done because the Halcyon couch does not allow for online rotational corrections during patient set‐up.

## 3 RESULTS

### 3.1 Retrospective EPID and kV CBCT image data collection

The EPID and kV CBCT images of patients treated between August 2019 and July 2020 were collected for head‐and‐neck cancer patients treated with VMAT on the Halcyon. This dataset consisted of 115 patients that were treated with 2541 treatment fractions. The patients were categorized by treatment site as listed in Table 1. The majority of patients were treated for laryngeal and oropharyngeal cancers.

**TABLE 1:** The EPID data collected for head‐and‐neck cancer patients categorized by treatment site.

Treatment and diagnoses sites	Patients (*n*)
Larynx	29
Oropharynx (p16‐)	27
Lip and oral cavity	24
Cervical lymph nodes & unknown primary tumors	7
Nasal cavity and sinuses	7
Hypopharynx	5
Nasopharynx	5
Salivary glands	5
HPV‐mediated (p16+) oropharyngeal cancer	2
Lacrimal gland	1
Nervous system (Misc.)	1
Orbit	1
Thyroid gland	1

### 3.2 EPID image analysis using the GDSA algorithm

For the 2541 fractions, the overall mean of the GDSA_μ_ values was 0.18% ± 0.66%. From Table 2, a total of 82 treatment fractions were delivered where the |GDSA_μ_| ≥ 3% and the majority of those were for laryngeal cancers (40 fractions). The overall mean and standard deviation of the GDSA_μ_ for the treatment sites where at least one patient treatment fraction had a |GDSA_μ_| ≥ 3% are listed in Table 3. The largest values of the mean of the GDSA_μ_ were for the nasopharyngeal, oropharyngeal, and laryngeal treatment sites. Most of these deviations occur during the later treatment fractions.

**TABLE 2:** Number of patients and total number of fractions per treatment site, and the number of fractions where | ΔGDSA_μ_ | ≥ 3%.

Tumor site	Patients	Fractions, *n* (% total)	*n* fractions | ΔGDSA_μ_ | ≥ 3%.
Larynx	29	641 (25.2%)	40
Oropharynx (p16‐)	27	597 (23.5%)	26
Lip and oral cavity	24	530 (20.9%)	2
Nasal cavity and sinuses	7	155 (6.1%)	7
Unknown primary H&N tumors	7	155 (6.1%)	0
Hypopharynx	5	110 (4.3%)	1
Nasopharynx	5	110 (4.3%)	3
Salivary glands	5	110 (4.3%)	3
HPV‐mediated (p16+) oropharyngeal cancer	2	44 (1.7%)	0
Lacrimal gland	1	22 (0.9%)	0
Nervous system (Misc.)	1	23 (0.9%)	0
Orbit	1	22 (0.9%)	0
Thyroid gland	1	22 (0.9%)	0

**TABLE 3:** The overall mean and standard deviation of GDSA_μ_ for the treatment sites that recorded any fraction where the | GDSA_μ_ | ≥ 3%.

Tumor site	*n* fractions | GDSA_μ_ | ≥ 3%.	Mean GDSA_μ_ (%)	STD of GDSA_μ_
Hypopharynx	1	0.63	1.34
Lip and oral cavity	2	0.27	0.89
Nasopharynx	3	0.72	1.09
Salivary glands	3	0.49	1.16
Nasal cavity and sinuses	7	0.19	0.82
Oropharynx (p16‐)	26	0.91	1.28
Larynx	40	0.77	1.27

**FIGURE 1:**
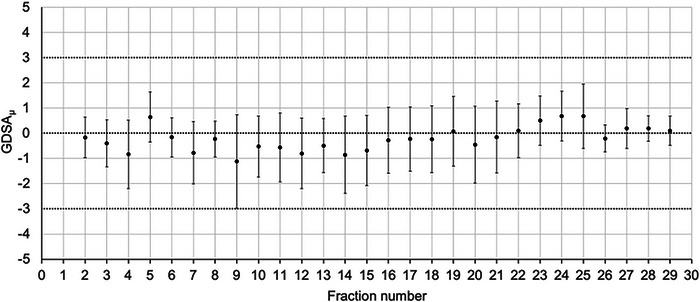
GDSA_μ_ versus fraction number for a nasal cavity cancer patient treated in 29 fractions. Error bars show the standard deviation.

There are a few general trends that can be identified when plotting the GDSA_μ_ as a function of fraction number (*n*). The first representative plot in Figure 1 is for a nasal cavity cancer patient. From this plot is apparent that there are minor deviations in the GDSA_μ_ between treatment fractions and it is generally considered to be stable. This is an indication that the tumor dose remains fairly consistent in each fraction, without major patient anatomical changes or daily setup variations.

**FIGURE 2:**
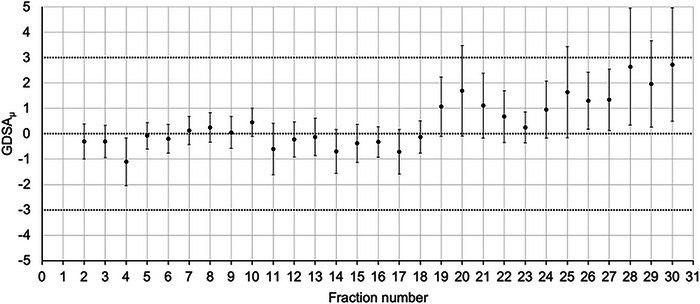
GDSA_μ_ versus fraction number for a maxillary sinus cancer patient treated in 30 fractions. Error bars show the standard deviation.

The GDSA_μ_ is plotted over 30 fractions for a maxillary sinus patient in Figure 2. The plot shows a general upwards trend from fraction 25 and is characteristic of the deviations seen in patients where continuous changes in weight and tumor shrinkage occur. In this scenario, the GDSA_μ_ does not exceed a 3% threshold; therefore, this patient was not re‐planned.

**FIGURE 3:**
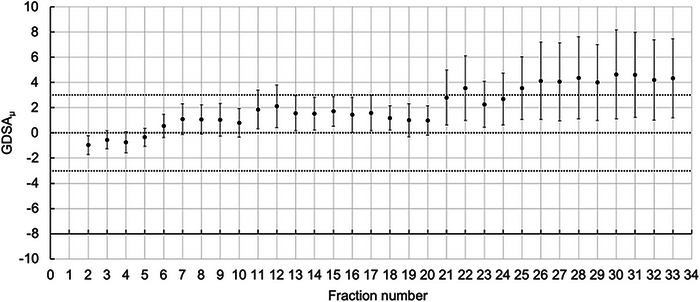
GDSA_μ_ versus fraction number for an oropharyngeal cancer patient treated in 33 fractions. Note the number of fractions where GDSA_μ_ ≥ 3%. Error bars show the standard deviation.

Figure 3 represents the plot of GDSA_μ_ for an oropharyngeal patient treated with 33 fractions. There is a general upwards trend from fraction 21 and the patient could have been replanned before major deviations (GDSA_μ_ ≥ 3%) occurred for fractions 24 to 33. If the GDSA had been implemented for daily verification, this could have been flagged on the day, and action could have been taken.

**FIGURE 4:**
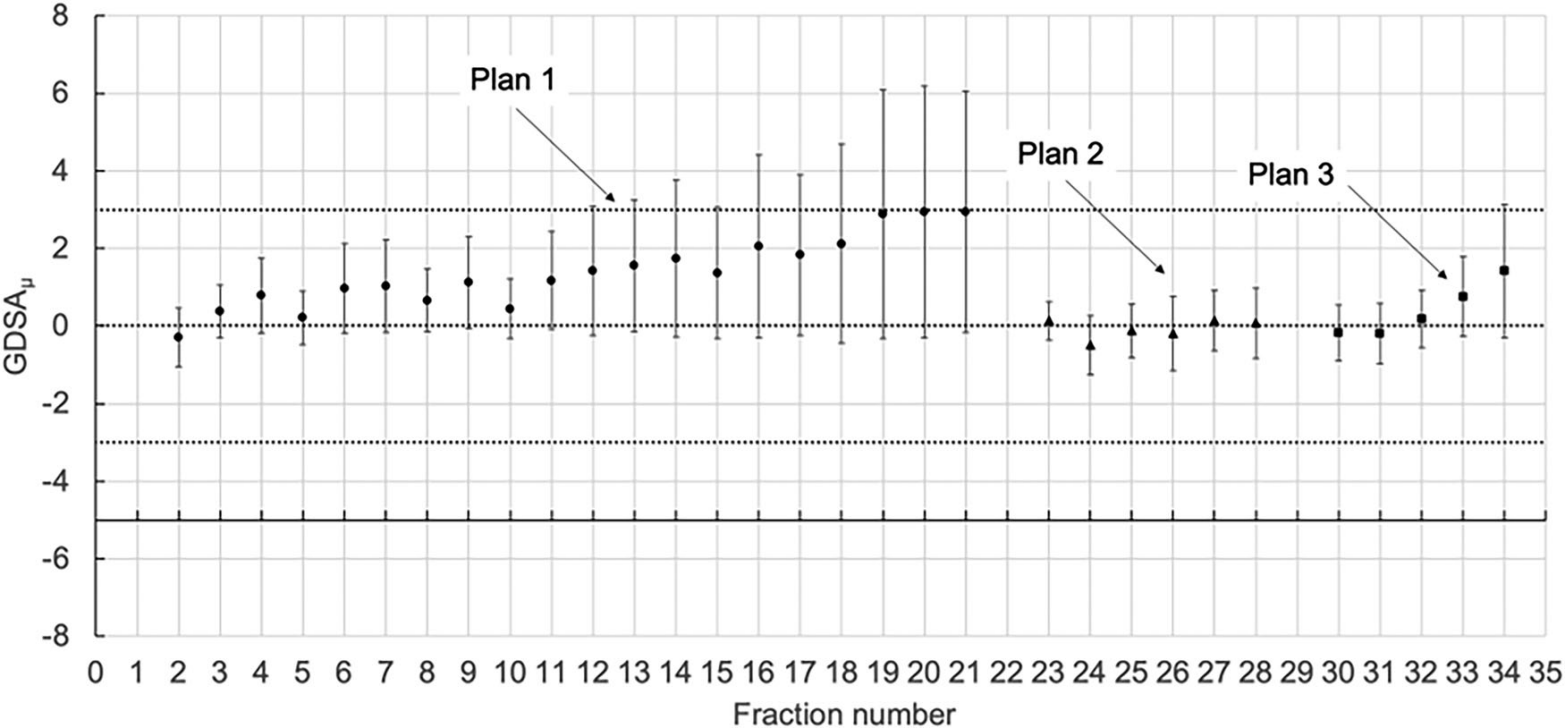
GDSA_μ_ versus fraction number for a nasopharyngeal cancer patient treated in 34 fractions. The first treatment fraction of the new treatment plan was used as a reference. Note the number of fractions where GDSA_μ_ ≥ 3%. Error bars show the standard deviation.

The GDSA_μ_ is plotted over 34 fractions for a nasopharyngeal patient in Figure 4. The patient was rescanned after fraction 21 and again after fraction 27. There is a gradual upwards trend in GDSA_μ_ up to fraction 21, which can be attributed to weight loss. Thereafter, the patient was rescanned and replanned, and continued treatment for a further seven fractions. Next, the patient was rescanned again, which pointed to issues radiotherapists had with immobilization and patient positioning during treatment. The patient completed treatment after receiving six more treatment fractions on the new treatment plan.

### 3.3 kV CBCT image analysis

The kV CBCT images were analysis in depth for the thirteen patients where the GDSA_μ_ ≥ 3% for at least one fraction. The maximum measured change in A‐P and lateral separation were $\Delta \;d_{A-P}=$ 3.91 cm and $\Delta \;d_{Lat}=$ 3.82 cm, respectively. The maximum effective separation change diameter was calculated to be $\Delta \;d_{eff}=$ 3.86 cm. For the subset of fractions where the GDSA_μ_ exceeded 3%, a moderate correlation (*R* ^2^ = 0.65) with the effective separation change diameter was found. The effective separation change diameter exceeded 1 cm for 92% of treatment fractions where the GDSA_μ_ exceeded the 3% threshold. The 1 cm effective separation change diameter threshold was applied for further analysis of patient data.

**FIGURE 5:**
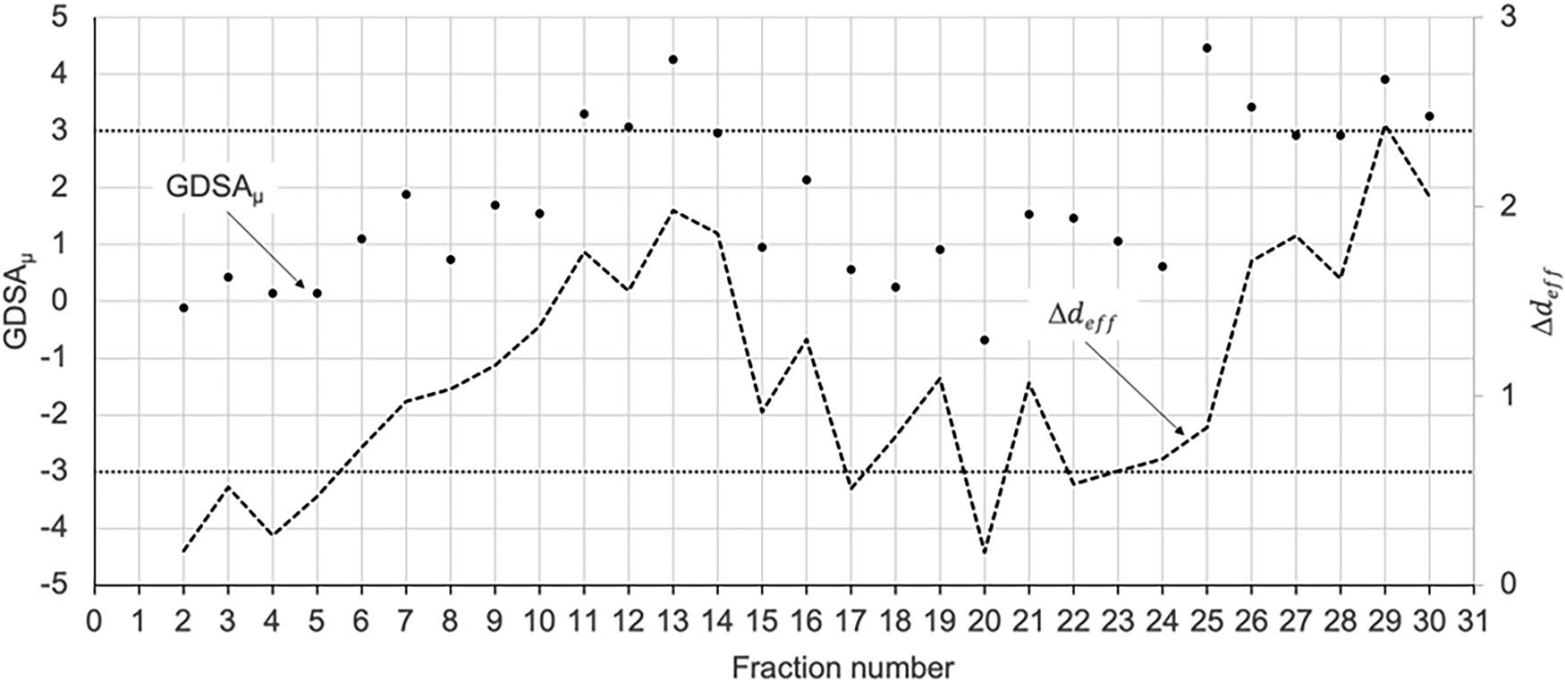
GDSA_μ_ and $\Delta d_{eff}$ (cm) versus fraction number for a laryngeal cancer patient treated in 30 fractions. Note the number of fractions where GDSA_μ_ ≥ 3%.

The GDSA_μ_ and $\Delta d_{eff}$ versus the number of fractions for a patient treated over 30 fractions are plotted in Figure 5. There is a moderate correlation (*R* ^2^ = 0.61) observed in the plot of the GDSA_μ_, versus the effective separation change diameter of the patient during treatment.

**FIGURE 6:**
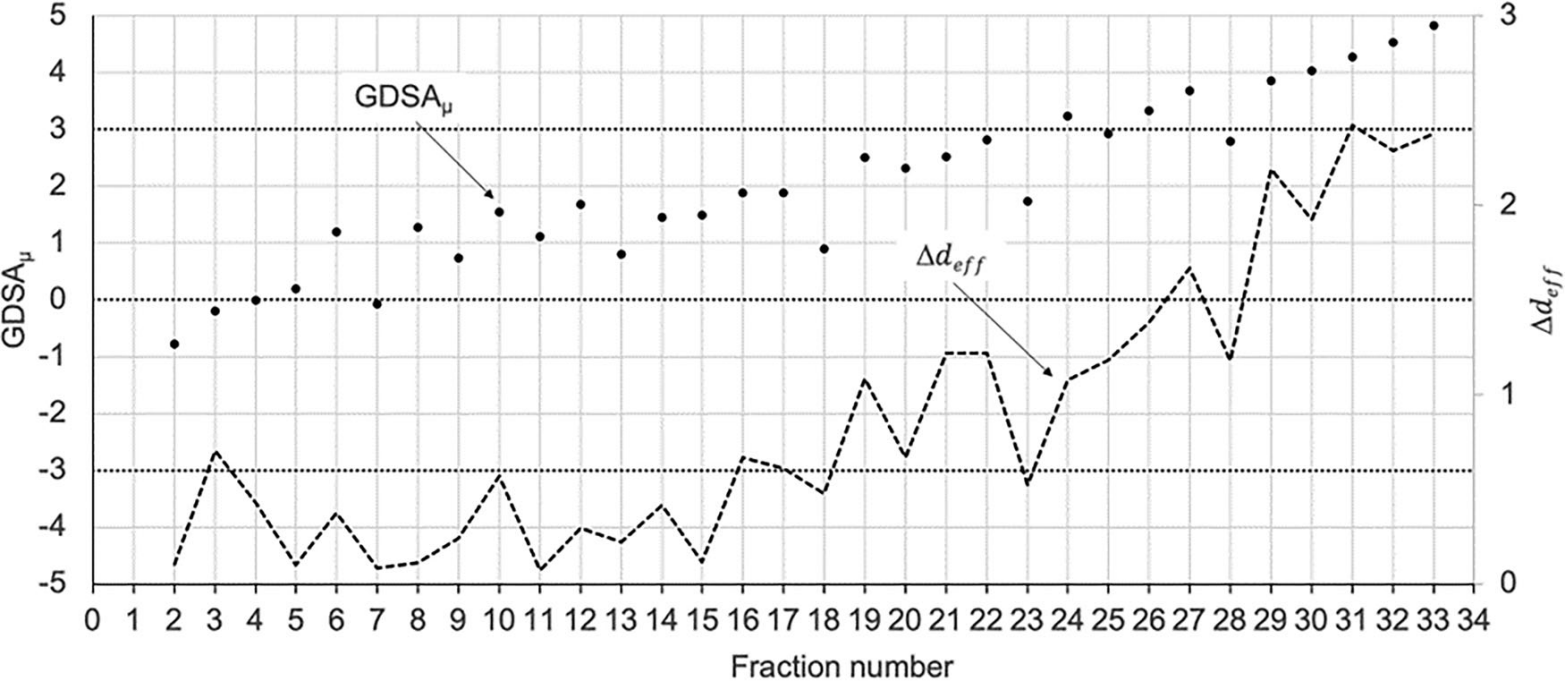
GDSA_[_ and $\Delta d_{eff}$ (cm) versus fraction number for an oropharyngeal cancer patient treated in 33 fractions.

Figure 6 is another plot of the GDSA_μ_ and $\Delta d_{eff}$ versus the number of fractions for a patient treated over 30 fractions. A relatively strong correlation was observed (*R* ^2^ = 0.82) in this plot. The GDSA_μ_ shows a continuous upward trend until the GDSA_μ_ ≥ 3% and the $\Delta d_{eff}\;>\;$1 cm.

The results for the rotational errors of the daily kV CBCT images for the thirteen patients where the GDSA_[_ ≥ 3% for at least one treatment fraction, were calculated and tabulated in Table 4. The maximum rotational errors were calculated as θ=3.90°, ζ=3.40°, and φ=2.59°, for pitch, roll, and yaw, respectively.

**TABLE 4:** The absolute maximum, mean, and standard deviation of pitch, roll, and yaw errors calculated for the thirteen patients where the | GDSA_[_ | ≥ 3%.

Rotational error	Maximum (degrees)	Mean (degrees)	STD
Pitch	3.90	0.90	0.89
Roll	3.40	0.45	0.51
Yaw	2.60	0.43	0.40

**FIGURE 7:**
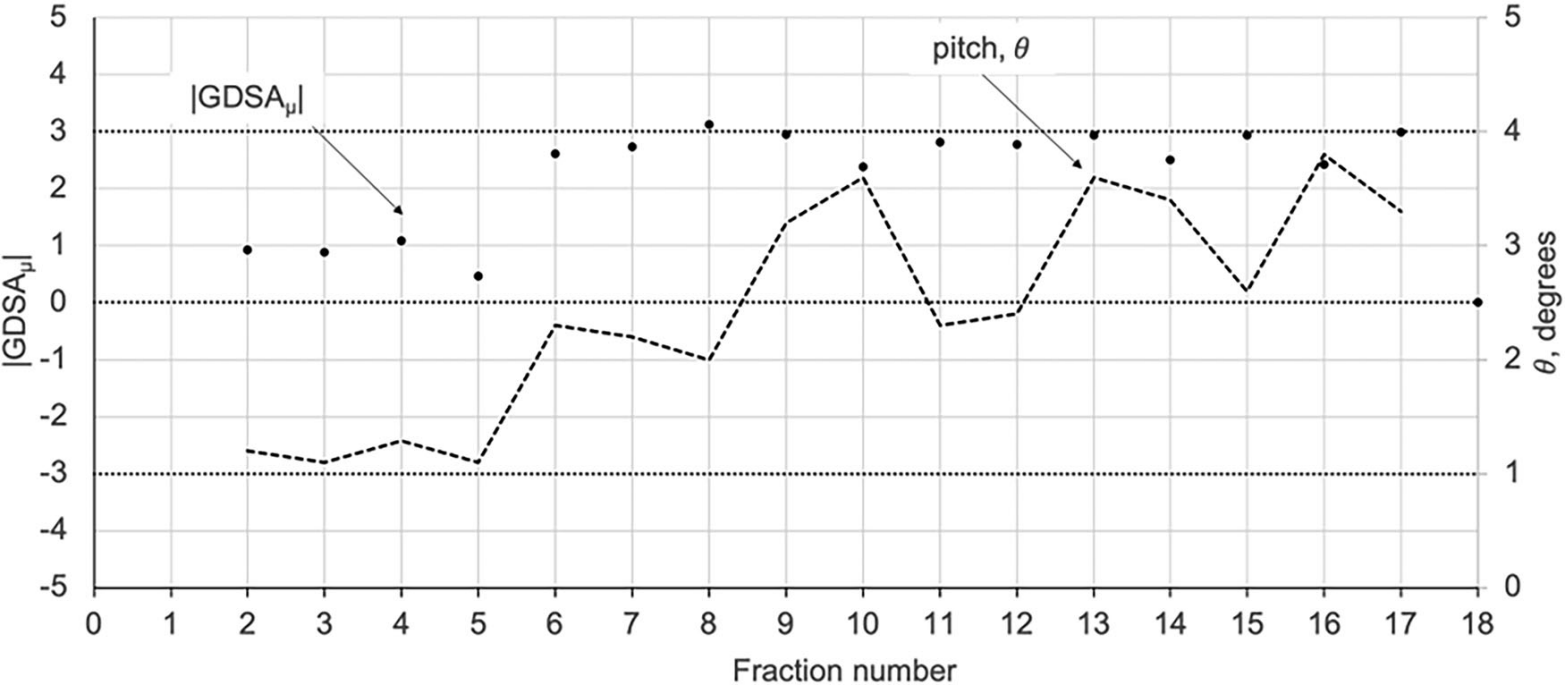
|GDSA_µ_| and pitch (θ, in degrees) versus fraction number for a laryngeal cancer patient treated in 18 fractions.

In patients where the GDSA_[_ ≥ 3%, but without changes in separation of $\Delta d_{eff}\;>\;$1 cm, there were errors in pitch between the reference and subsequent fractions. Figure 7 shows an example plot of the |GDSA_[_| and pitch (θ) versus treatment fractions.

The overall correlation between the GDSA_µ_ and pitch (θ), for the subset of treatment fractions where the GDSA_µ_ exceeded 3%, but $\Delta d_{eff}\;$< 1 cm, was found to be weak (*R* ^2^ = 0.37).

## 4 DISCUSSION

The overall mean of the GDSA_µ_ was 0.18% ± 0.66% which is comparable to the results published by Steers et al.^16^ for head‐and‐neck cancer patients treated in their institution. The GDSA_µ_ exceeded 3% for 82 of 2541 treatment fractions; of these 82, over 48% were patients treated for laryngeal tumors. Plotting the GDSA_µ_ versus treatment fractions showed a variety of trends that are synonymous with head‐and‐neck radiotherapy treatments. Firstly, it is shown that many head‐and‐neck cancer patients continuously lose weight during treatment, and weight‐loss causes significant changes in PTV D_mean_ during the later fractions of treatment. Secondly, it was found that the effective separation change diameter exceeds 1 cm for patients where the GDSA_µ_ exceeds 3%. Sun et al.^20^ found that uniform body changes less than 1 cm were unlikely to warrant further assessment due to changes in delivered dose. Similarly, Chen et al.^21^ found that the dose delivered to the PTV significantly increased by 2%–3% for a 2–5 mm change in body contour. This is an important finding, as it is much easier to implement the GDSA_µ_ than to measure the effective separation change diameter per fraction for every patient on every treatment day.

For patients were the |GDSA_µ_| exceeded 3%, but without an effective separation diameter change of more than 1 cm, significant rotational errors were found. Figure 7 shows that there is a possible relationship between |GDSA_µ_| values and the magnitude of pitch errors in patient setup. The mean and standard deviation in pitch, roll, and yaw rotational errors listed in Table 4 (0.90° ± 0.89°, 0.45° ± 0.51° and 0.43° ± 0.40°, respectively), correspond to the 0.96° ± 1.99°, −0.62° ± 1.44°, and −0.17° ± 0.97° found by Zhang et al.^22^ for head‐and‐neck patients. Finally, Guckenberger et al.^23^ showed that rotational errors may be of clinical significance for patients with elongated, non‐spherical target volumes and steep dose gradients. Almost half of the GDSA_µ_ failures in this work were for larynx patients, which tend to be elongated tumors.

The major limitation of applying the GDSA_µ_ to the patient dataset, is that the data from Fraction 1 is used as the reference. This means that potential set‐up errors and patient anatomy changes can influence the reference fraction. The effect of this can be minimized by means of a mandatory offline review of the first kV CBCT image and matching by the radiation oncologist. This will allow the assessment of anatomy changes and set‐up geometry.

The GDSA can easily be incorporated into the clinical workflow. The suggested workflow includes the automatic export of EPIDs from the record‐and‐verify system, as well as GDSA analysis, after each treatment day. The user would then receive an e‐mail notification should the GDSAµ exceed the 3% user‐selectable tolerance. This will allow the radiation oncologist and medical physicist to review the patient treatment using the offline image review and decide on the appropriate cause of action.

## 5 CONCLUSIONS

The GDSA_µ_ was able to show clear trends between patient weight changes and changes in the PTV D_mean_. For patients with minimal weight changes, the pitch was the highest calculated rotational error. However, more data will be needed to fully assess the sensitivity of the GDSA_µ_ for errors in pitch. The GDSA_µ_ algorithm is easily implementable and has the means to improve resource allocation in resource‐constrained environments. The current data will also be used as a baseline in the department's QA program.

## AUTHOR CONTRIBUTIONS

Christoffel Jacobus van Reenen designed the study, collected, analyzed the data, and wrote the manuscript. Christoph Jan Trauernicht and Casey Bojechko oversaw data analysis and interpretation, and helped write the final manuscript. All authors approved the final manuscript version.

## CONFLICT OF INTEREST STATEMENT

The authors declare no conflicts of interest.
